# Aberrant Expression of BUB1B Contributes to the Progression of Thyroid Carcinoma and Predicts Poor Outcomes for Patients

**DOI:** 10.7150/jca.68408

**Published:** 2022-04-18

**Authors:** Hai-chao Yan, Cheng Xiang

**Affiliations:** Department of Thyroid Surgery, The Second Affiliated Hospital of Zhejiang University College of Medicine, Hangzhou 310009, Zhejiang, China.

**Keywords:** thyroid carcinoma, BUB1B, high expression, prognosis, mechanism

## Abstract

**Objective:** This study aimed to clarify the function and potential mechanism of BUB1B in THCA.

**Methods:** Expression of BUB1B in THCA was firstly determined, and its important prognostic value was then demonstrated. The potential mechanism was initially predicted by KEGG analysis. To explore the specific function of BUB1B in THCA, we used lentivirus infection to knock down the BUB1B, and then performed flow cytometry, colony formation, transwell, and wound-healing assays. Related protein expression was detected through western blotting. Additionally, we predicted the BUB1B-regulated pathways involved in THCA by GSEA analysis.

**Results:** BUB1B expression was highly increased in THCA tissues relative to normal controls. We further found that BUB1B was essential for tumor cell proliferation, and BUB1B high expression predicted a shorter PFS time of THCA patients. More importantly, Cox regression determined the BUB1B as an independent prognostic factor for PFS in THCA. BUB1B was initially found to participate in the cell cycle pathway from KEGG analysis. Unexpectedly, we did not detect the disturbing effect on the cell cycle distribution of THCA cells with BUB1B knockdown. But, BUB1B knockdown inhibited the proliferation, invasion, and migration of THCA cells, as well as increased apoptotic cells, and the results were further confirmed by western blotting. Through GSEA analysis, we predicted a positive correlation between BUB1B and metastasis-related pathways such as mTOR and NF-kappa B signaling pathways.

**Conclusions:** Present study identified BUB1B as a promising clinical prognostic factor in THCA, as well as a potential novel therapeutic target for cancer treatment.

## Introduction

Thyroid carcinoma (THCA) is the most prevalent malignancy of the endocrine system, and its incidence is gradually increasing worldwide 1. Although the 10-year survival rate of THCA patients was larger than 95%, 5-20% of patients were still subject to disease recurrence and distant metastasis 2. Especially, the most aggressive type of THCA, anaplastic thyroid carcinoma showed a poor prognosis with a 39% survival rate at 1 year 3. With the development of sequencing technology, research on THCA is progressing at the genetic level. It has been found that many genes were related to the occurrence, development, and prognosis of THCA. Zhao et al. performed a meta-analysis of case-control study and found a strong relationship between elevated concentrations of tumor necrosis factor-alpha (TNF-α) and THCA 4. Lv et al. identified 4 methylation-driven genes in THCA with independent prognostic value, which provided new insight into molecular mechanisms of THCA 5. Ma et al. revealed 2 RNA binding proteins were independently associated with the prognosis of patients with THCA, and significantly affected cancer cell proliferation and migration 6. Zhao et al. found that down-regulation of glucose-regulated protein GRP78 could significantly inhibit the metastatic and proliferative ability of THCA cells 7. Cyclin E2 (CCNE2), exerting an important role in controlling the transition of quiescent cells into the cell cycle, regulating the G1/S transition, and promoting DNA replication, which has been proved to lead to carcinogenesis and the development of THCA 8. There is an urgent need for identifying more specific biomarkers to reveal the pathogenesis of THCA.

It is commonly known that the cell mitosis process plays a crucial role in maintaining body functions. Spindle assembly checkpoint (SAC) has become an important monitor in the process of cell division, as it plays a vital role in arresting cell division until the precise chromosome segregation is ensured during mitosis and meiosis to maintain genomic stability 9. Abnormal expression of SAC proteins can lead to the instability of chromosomes and promote cell malignant transformation. BUB1 mitotic checkpoint serine/threonine kinase B (BUB1B) is a key regulator of the SAC family 10. Growing evidence has demonstrated that aberrant expression of BUB1B was highly involved in the tumorigenesis and development of various tumors. Scintu et al. demonstrated an increase of BUB1B transcripts in the majority of ductal breast carcinoma samples, and BUB1B mRNA levels correlated with intrachromosomal instability 11. A high expression of BUB1B was also found in prostate cancer, indicating that BUB1B was essential for efficient tumor cell proliferation and correlated with poorer patient outcomes 12. In addition, BUB1B has been identified as the top-scoring glioblastoma lethal kinase, and knockdown of BUB1B inhibited the expansion of brain tumor-initiating cells isolates 13. It followed that BUB1B significantly participated in the tumor progression.

From the aspect of clinical value, a meta-analysis has demonstrated that BUB1B was a significant biomarker for a poor prognosis and poor clinicopathological outcomes in patients with lung adenocarcinoma 14. BUB1B has been determined as a useful prognosis biomarker in several human cancers such as colon cancer 15, colorectal cancer 16, gastric cancer 17, and hepatocellular carcinoma 18. In addition, combined expression of BUB1B and others genes were also proved to be a strong predictor of overall survival in adult adrenocortical tumors 19. These researches suggested the importance of BUB1B in tumor progression and significant prognostic value on patient survival. At present, only two studies by bioinformatics analysis revealed BUB1B as a hub gene in anaplastic thyroid carcinoma 20, 21. However, the detailed function and role of BUB1B in THCA have not been revealed up to now.

In this study, we discovered that BUB1B was highly expressed in THCA tissue compared with normal tissue. Kaplan-Meier and Cox regression analyses uncovered the independent prognostic value of BUB1B high expression for predicting a shorter progression-free survival in THCA patients. We further determined that BUB1B was involved in THCA progression by influencing the proliferation, apoptosis, invasion, and migration of THCA cells. Our study initially and systematically demonstrated the importance of BUB1B in THCA and revealed the possible mechanism associated with BUB1B in THCA.

## Materials and Methods

### Expression analysis and verification of BUB1B in THCA

We firstly evaluated the mRNA expression of BUB1B in THCA and normal samples by analyzing the mRNA expression profile of THCA patients, which was obtained from the TCGA database. The protein expression of BUB1B and its subcellular location in THCA were further detected by immunohistochemistry and immunofluorescence. We obtained thyroid tissue samples consisting of the tumor tissues and normal tissues from The Second Affiliated Hospital of Zhejiang University College of Medicine. All specimens were handled and made anonymous according to ethical and legal standards. Our study obtained the consent of the Ethics Committee of the The Second Affiliated Hospital of Zhejiang University College of Medicine before the research began.

### Prognosis and genetic alteration analysis of BUB1B inTHCA

We then explored the prognostic value of BUB1B in THCA patients based on TCGA data. The inclusion criteria: (a) samples diagnosed as thyroid cancer; (b) samples with mapped clinical information and gene expression matrix; (c) samples with complete clinical information including survival time, survival status, age, and gender. The exclusion criteria: (a) normal tissue samples; (b) samples without complete clinical information; (c) samples with no expression value; (d) samples with bias in expressional value. This study evaluated the prognostic impact of BUB1B expression on the overall survival (OS) and progression-free survival (PFS) of THCA patients via Kaplan-Meier survival analysis. Restrict survival analysis was performed based on the patient's age, gender and clinical stage. Cox regression analysis was used to predict the independent prognostic value of BUB1B in THCA. We also explored the mRNA expression and prognostic impacts of BUB1B in various human cancers in the GSCA database. In addition, genetic alteration of genes is one of the causes affecting cancer development. Hence, we further assessed the genetic alteration of BUB1B in THCA (TCGA, Firehose Legacy) in the cBioportal database which contained 397 samples with complete genetic alteration data, and explored the somatic mutation of BUB1B in the COSMIC database.

### Combined effect exploration of BUB1B and TOP2A in THCA

To further verifiy the importance of BUB1B in clinical, we explored the combined effects of BUB1B with other gene on the prognosis of THCA patients. From cBioportal database, Topoisomerase (DNA) II Alpha (TOP2A) showed the largest correlation with BUB1B. We then detected the relationship between them in TIMER database and western blotting. The mRNA expression of TOP2A in THCA was ebvaluated in GSCA database. The prognostic effect of TOP2A on the PFS of THCA patients were evaluated through Kaplan-Meier analysis using the TCGA-THCA data. Independent prognostic value of TOP2A in THCA was assessed through Cox regression analysis. Subsequently, we assessed the combined the prognostic impacts of 2 genes on the PFS of THCA patients.

### Enrichment analysis on BUB1B co-expressed genes

For revealing the potential mechanism of BUB1B in THCA, we firstly obtained the co-expressed genes with BUB1B in THCA through the cBioportal database. The genes with absolute correlation coefficient>0.5 and P<0.05 were selected for the further enrichment analysis containing GO annotation and KEGG pathway prediction. GO annotation analysis contained biological process (BP), cellular component (CC), and molecular function (MF) exploration. KEGG analysis was used to predict the potential pathways associated with these genes. We used the clusterProfiler R package to perform the enrichment analysis.

### Immunofluorescence assay

The THCA cell slides were fixed with 4% paraformaldehyde for 20 min and incubated with 5% BSA solution at room temperature for 90 min. The diluted primary and secondary antibodies were successively taken to incubate the slides. The slides were then washed with PBS, and DAPI was added to stain the nucleus for 5 min. After rinsing slides with PBS, phalloidine was diluted with PBS and added to slides, followed incubation for 20 min. Finally, the immunofluorescence images were obtained.

### Cell culture and plasmid transfections

Two human cell lines (BCPAP and TPC-1) of human thyroid carcinoma was obtained from BeNa Technology (Hangzhou)were used for conduct experiment. The BCPAP and TPC-1 cells were cultured in Dulbecco's modified Eagle's medium DMEM (Hyclone) supplemented with 10% fetal bovine serum (FBS), 100 U/ml penicillin, and 100 g/ml streptomycin at 37 ℃ with 5% CO_2_. Three small interfering RNA (siRNA) were designed and constructed for inhibiting BUB1B/TOP2A expression (siRBUB1B/siRTOP2A) (**Table.1**). Before transfection, we mixed 2 μl Lipofectamine 2000 and 50 μl Opti-MEM as solution 1 and mixed 100 nMsiRNA and 50 μl Opti-MEM as solution 2. Then, the THCA cells were transfected for 24-48 h with miscible liquids of solution 1 and solution 2. The knockdown efficiency was determined by western blot.

### Cell viability detection

After transfection for 24 and 48h with siRNA, the cells were harvested and counted on a hemocytometer. The MTT assay was utilized to assess cellular proliferative potential based on the manufacturer's protocol. In brief, we planted the stable cell lines into 96-well plates (1000-10000 cells/well). After incubation for 3 hours at 37°C in the incubator described above, we measured the absorbance (570 nm) of each well and obtained the optical density (OD) value.

### Apoptosis assay

We used the Annexin V-FITC/propidium iodide (PI) Apoptosis Detection Kit to identify apoptotic cells based on the manufacturer's protocol. The cells were firstly collected with trypsin without EDTA and then rinsed with PBS. The 100 μl cell suspension was placed to a 5 ml tube, and then 5 μl Annexin V/FITC solution was added. After adding 10 μl PI solution (20 μg/ml) and 400 μl PBS, the apoptosis level of cell samples was immediately detected by the flow cytometry.

### Cell cycle assay

The cell cycle distribution was detected by flow cytometry. We first digested the stable cell lines with trypsin for 2 min, then added DMEM to the culture dish to stop the digestion. We collected the cell suspension and centrifuged them at 400 g for 5 min. After rinsing the cell lines, we discarded the PBS and first fixed the cell with 750 μl of 75% ethyl alcohol for 18-24h at 4°C. We then collected the cell lined after centrifugation at 300 g for 5 min. Subsequently. the cells were incubated with 2.5μl RNase and 25 μl PI solution for 30 min at room temperature. The cell cycle distribution was detected within 24 h.

### Colony formation assay

We seeded the cell lines into 6-well plates. After culturing all cells with DMEM for 10 days, we discarded the culture media and washed the cells with PBS two times. Then we fixed the cells with 0.8ml methanol for 20 min. Finally, we stained the cells with 0.8ml crystal violet dye solution and observed the proliferating colonies with an inverted microscope.

### Transwell invasion assay

The 100μl Matrigel solution was firstly spread out in the upper chambers, and the cell suspension (100 μL) was then added to the upper chambers. The lower chamber contained 500 μl 10% fetal bovine serum used as a chemoattractant. After incubation for 24 h at 37°C, cells were fixed with methanol for 20 min and then stained with crystal violet dye solution for 40 min. Finally, the images for transwell invasion were obtained by an inverted microscope.

### Wound-healing assay

The scratch wound-healing motility assay was performed to evaluate the migration ability of THCA cells. The transfections with BUB1B or negative control plasmids were carried out when the cell reached 80%-90% confluence. The 24 h and 48 h after the transfection, a scratch was made with a 10 μL pipette tip. The cells were then returned to the incubator until the indicated time. Representative sites were photographed, and the cells that migrated from the wound edge were counted at each time point.

### Western blotting analysis

The proteins were extracted for Western blot analyses after 24 and 48h transfection. Western blotting was performed on the basis of the manufacturer's instructions and related information as previously described. The antibodies used for western blotting in this study are described in **Table 2**.

### Statistical analysis

The data in this study were analyzed with SPSS 23.0. All experiments were repeated three times and related data were expressed as mean±SD. Statistical differences of quantitative data between groups were evaluated using the Student's *t*-test or one-way analysis of variance (ANOVA). The qualitative data between groups was compared with χ^2^ test. Survival difference between groups was compared with the Kaplan-Meier method and log-rank test. Independent prognostic analysis was performed with Cox regression. Correlation between 2 genes was detected with the spearman method. P<0.05 was regarded as the statistical significance.

## Results

### BUB1B is highly expressed in THCA and associated with tumor progression

**Figure 1:**
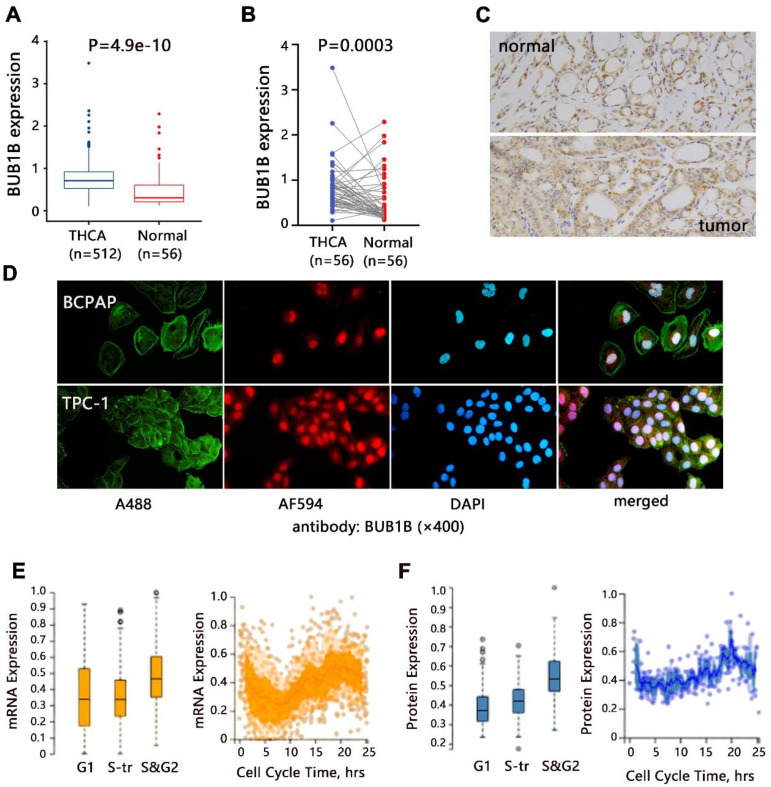
Expression analysis of BUB1B in THCA. **(A)** mRNA expression of BUB1B in normal and THCA samples (TCGA). The two-sample *t* test was used to compare the difference between 2 groups. **(B)** mRNA expression of BUB1B in paired normal and THCA samples (TCGA). The paired* t* test was used to compare the difference between 2 groups. **(C)** The protein expression of BUB1B in normal and tumor tissues by immunohistochemistry method. **(D)** Location verification of BUB1B by immunofluorescence. **(E)** BUB1B mRNA expression across cell cycle (HPA). **(F)** BUB1B protein expression across cell cycle (HPA). Abbreviation: THCA, thyroid carcinoma; BUB1B, BUB1 mitotic checkpoint serine/threonine kinase B.

We firstly evaluated the mRNA expression of BUB1B in normal and THCA tissues based on TCGA-THCA data. The differential expression analysis showed that BUB1B mRNA was highly expressed in THCA tissues compared with that in normal tissues (**Fig. 1A**, P<0.001), and BUB1B high expression in THCA was confirmed through paired samples analysis as well (**Fig. 1B**, P=0.003). In addition, the protein expression of BUB1B in clinical normal and cancer samples was detected by the immunohistochemistry method and presented in **Fig. 1C**. From the HPA database in our previous investigation, we found that BUB1B was mainly located at cytosol (data not shown), and its subcellular location in THCA cells was also detected *in vitro* in our study by immunofluorescence (**Fig. 1D**). In addition, we found BUB1B variation in the transcript (**Fig. 1E**) and protein (**Fig. 1F**) expression correlated to the cell cycle.

**Figure 2:**
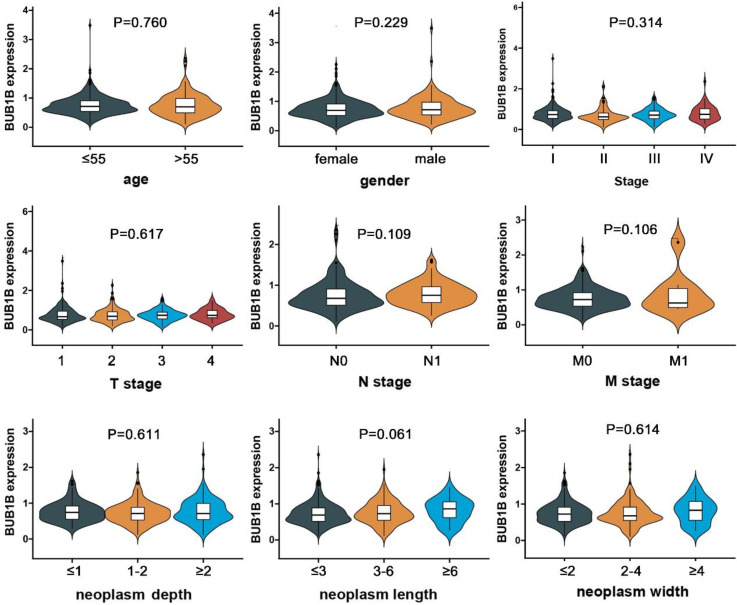
Association of BUB1B mRNA expression with clinical clinicopathological features of THCA patients. Abbreviation: THCA, thyroid carcinoma; T, tumor topography; N, lymph node metastasis; M, distant metastasis; BUB1B, BUB1 mitotic checkpoint serine/threonine kinase B; The two-sample *t* test was used to compare the difference between 2 groups, and one-way ANOVA was used to compare the difference among large than 2 groups.

We also explored the correlation between BUB1B mRNA expression and the clinical futures of patients based on TCGA data. All patients were assigned into low and high expression groups according to BUB1B median expression, then we assessed the expression difference of BUB1B under different clinical characteristics by qualitative analysis (**Table 3**). We subsequently evaluated the detailed expression level of BUB1B in various clinical futures of patients by quantitative analysis, and the results showed that patient's age, gender, T stage, N stage, M stage, clinical stage, neoplasm depth, length, and width did not influence BUB1B mRNA expression (**Fig. 2**).

**Figure 3:**
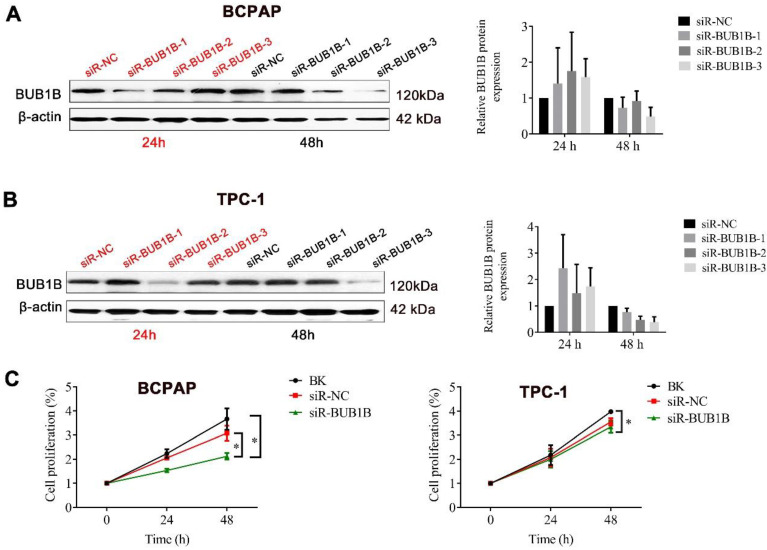
The effect of BUB1B on tumor cell growth. The **(A)** and **(B)** indicated the knockdown efficiency of BUB1B in THCA cell lines through western blot. **(C)** Cell proliferation in THCA cell lines with BUB1B knockdown was assessed with MTT assay. The two-sample *t* test was used for difference comparison between 2 groups (BK vs siR-NC, siR-NC vs siR-BUB1B). Abbreviation: THCA, thyroid carcinoma; BUB1B, BUB1 mitotic checkpoint serine/threonine kinase B.

To investigate the effects of abnormal BUB1B expression in THCA cells, siRNAs specific for BUB1B were used to deplete the expression of BUB1B in BCPAP and TPC-1 cells, for 24 and 48 h. The knockdown efficiency was verified by western blot (**Fig. 3A, 3B**), founding that cell viability was significantly decreased in the BUB1B-knockdown group compared with other groups after 48h knockdown (**Fig. 3C**). These results suggested that BUB1B might be involved in the growth of THCA cells. On account of possessing the highest knockdown efficiency in 2 type cells, the siR3 was chosen for all subsequent experiments.

**Figure 4:**
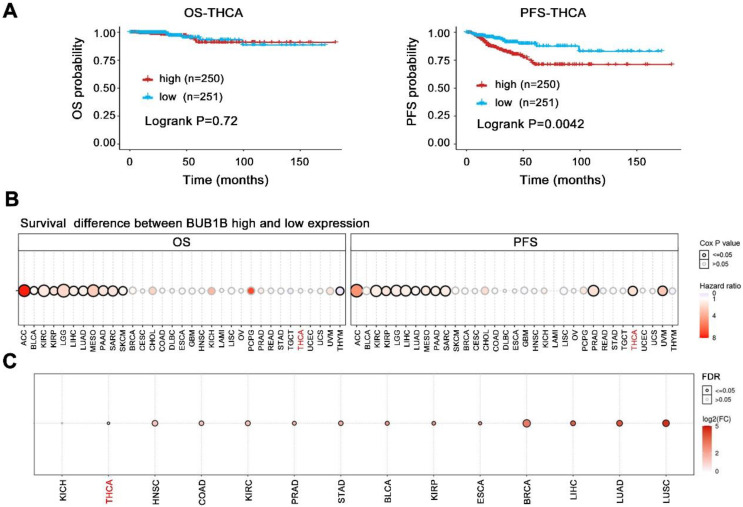
Effects of BUB1B mRNA expression on the prognosis of patients. **(A)** Kaplan-Meier survival analysis on BUB1B in terms of OS and PFS in THCA. The survival difference between 2 groups was compared with log-rank test. **(B)** Survival difference between BUB1B high and low expression groups in 33 cancer types. The bubble plot presented the hazard ratio and Cox p-value through bubble color and size. The row is the gene set symbol, and the column is the selected cancer types. The bubble color from blue to red represents the hazard ratio from low to high, bubble size is positively correlated with the Cox P-value significance. The black outline border indicates Cox P-value≤0.05. **(C)** The mRNA expression of BUB1B in various human cancers. Abbreviation: THCA, thyroid carcinoma; OS, overall survival; PFS, progression-free survival; BUB1B, BUB1 mitotic checkpoint serine/threonine kinase B.

The above results have indicated BUB1B high expression in THCA and its influence on tumor cell growth, we further evaluated its prognostic impact on THCA patients. The BUB1B expression did not influence the overall survival of patients (**Fig. 4A**, P=0.72), but THCA patients with higher BUB1B expression were characterized by a shorter progression-free survival (P=0.0042). Further restrict survival analysis based on the patient's age, gender, and the clinical stage was performed and presented in **Table 4**.

Additionally, among 33 cancer types, BUB1B higher expression caused a worse overall survival and shorter progression-free survival time in one-third of types (**Fig. 4B,** all HR>1, all P<0.05). The favorable prognostic impact of BUB1B in 33 cancer types was not observed. Even more, unfortunately, BUB1B was highly expressed in almost all cancers types including THCA (**Fig. 4C**). These results suggested that BUB1B acted as an oncogene and its abnormal high expression exerted a vital influence on cancer patient's survival including THCA.

In addition, the Cox proportional hazards regression model was used for univariate and multivariate analyses (**Table 5**). The univariate and multivariate analyses indicated that upregulation of BUB1B was an independent predictor for shorter progression-free survival in THCA (all HR>1, all P<0.05). However, the upregulation of BUB1B could not predict shorter overall survival both in univariate and multivariate analyses (all P>0.05).

Taken together, our results indicated that BUB1B expression was upregulated in THCA and significantly associated with tumorigenesis. BUB1B high expression could independently predict a shorter progression-free survival of THCA patients. The above results suggested that BUB1B was probably involved in the tumorigenesis and progression of THCA and a likely prognostic biomarker for THCA.

**Figure 5:**
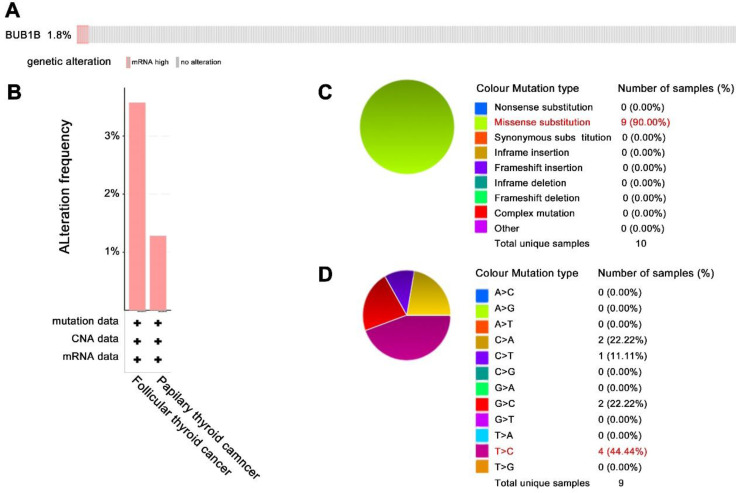
The genetic alteration of BUB1B in THCA.** (A)** Genetic frequency of BUB1B among 397 THCA samples (cBioportal). **(B)** BUB1B genetic alteration based on detailed cancer type. **(C)** Overview of the types of mutation observed. **(D)** Breakdown of the observed substitution mutations. Abbreviations: THCA, thyroid carcinoma; BUB1B, BUB1 mitotic checkpoint serine/threonine kinase B.

In addition to aberrant expression, mutation of BUB1B can cause aneuploidy, thus affecting cancer development. Hence, we also explored BUB1B genetic alteration in THCA. The 1.8% patients among 397 samples appeared genetic alteration with characteristics of mRNA high expression (**Fig. 5A**). BUB1B alteration based on cancer types was shown in **Fig. 5B**. The main mutation type of BUB1B in THCA was missense substitution (**Fig. 5C**). Single nucleotide variation for missense substitution was presented as T>C (**Fig. 5D**).

### Combined effect of BUB1B and TOP2A in THCA

**Figure 6:**
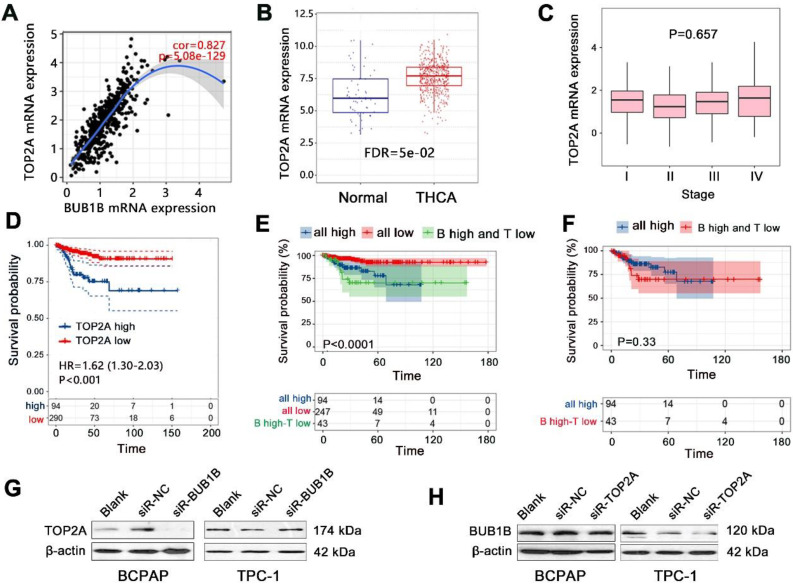
Combined effect exploration of BUB1B and TOP2A in THCA. **(A)** Spearman correlation analysis between BUB1B and TOP2A mRNA expressions in THCA (TIMER). **(B)** The mRNA expression of TOP2A in normal and THCA samples.** (C)** Correlation of TOP2A mRNA expression with clinical stage in THCA.** (D)** Effect of TOP2A expression on PFS time of THCA patients. **(E)** Prognostic impact analysis on THCA patient's PFS with the combination of BUB1B and TOP2A. **(F)** PFS survival time difference between 2 groups. **(G)** TOP2A protein expression with BUB1B knockdown. **(H)** BUB1B protein expression with TOP2A knockdown. Abbreviation: THCA, thyroid carcinoma; PFS, progression-free survival; FDR, false discovery rate; BUB1B, BUB1 mitotic checkpoint serine/threonine kinase B; TOP2A, Topoisomerase (DNA) II Alpha; all high: BUB1B high expression-TOP2A high expression; all low, BUB1B low expression-TOP2A low expression; B high-T low, BUB1B high expression-TOP2A low expression; NS, no significance.

The previous study has indicated the significant prognostic value of combined expression of BUB1B with several genes in human cancers; this study also explored the combined prognostic value of BUB1B with important genes in THCA. We detected all co-expressed genes of BUB1B in THCA, finding that TOP2A showed the largest positive correlation with BUB1B (**Fig.6A**). We then explored the interaction between them by western blotting, finding that TOP2A protein expression was downregulated in THCA cells with BUB1B knockdown (**Fig. 6G**). Knockdown of TOP2A also caused the BUB1B expression to decrease (**Fig. 6H**). Survival analysis firstly showed that TOP2A high expression shortened the progression-free survival time of THCA patients (**Fig. 6D**, P<0.001). Expression analysis showed that TOP2A was highly expressed in THCA tissue compared with normal tissue (**Fig. 6B**), but its expression did not correlate with the clinical stage of THCA (**Fig. 6C**). These results indicated TOP2A as an oncogene in THCA. We further assessed the importance of TOP2A in THCA progression by Cox regression analysis, finding that TOP2A expression was an independent prognostic factor for progression-free survival of THCA patients (**Table 6**).

We further explored the combined prognostic impacts of BUB1B and TOP2A on PFS of THCA patients. Compared with all low-expression group, all high expression of 2 genes decreased the survival probability of patients (**Fig. 6E**). No difference in survival time was observed between all high-expression groups and the group with BUB1 high expression-TOP2A low expression (**Fig. 6F**). It followed that consistent low expression of BUB1B and TOP2A exerted a vital influence on the patient's survival. The detailed prognostic impacts on THCA patient needs further investigation in a clinical cohort.

### Enrichment analysis on BUB1B

**Figure 7:**
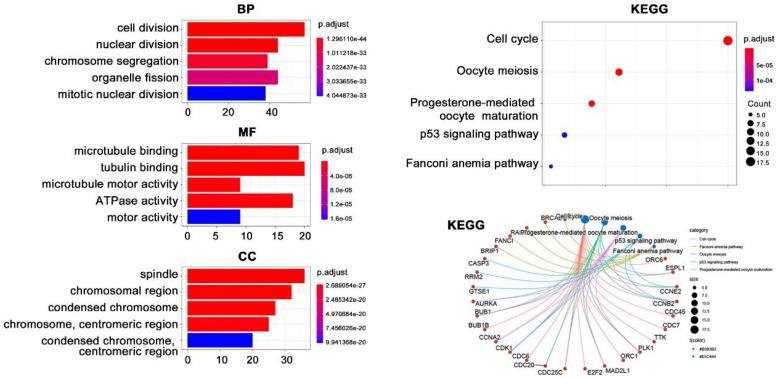
Functional enrichment analysis on BUB1B co-expressed genes in THCA. Abbreviations: THCA, thyroid carcinoma; BUB1B, BUB1 mitotic checkpoint serine/threonine kinase B; BP, biological process; MF, molecular function; CC, cellular component; KEGG, Kyoto Encyclopedia of Genes and Genomes.

Due to the importance of BUB1B in THCA, we next performed enrichment analysis to reveal its potential function (**Fig. 7**). A total of 123 co-expressed genes of BUB1B in THCA were firstly obtained by setting absolute spearman's correlation>0.5 and P<0.05. Subsequent GO analysis showed that these 123 genes were mainly located at spindle and chromosome (CC), and participated in cell division, nuclear division, and chromosome segregation processes (BP). The molecular function mainly referred to tubulin-related binding (MF). KEGG pathway analysis indicated that these genes possibly correlated with cell cycle and oocyte meiosis. Further information showed that BUB1B referred to the cell cycle pathway.

### BUB1B influences proliferation, migration, and apoptosis in THCA cells

**Figure 8:**
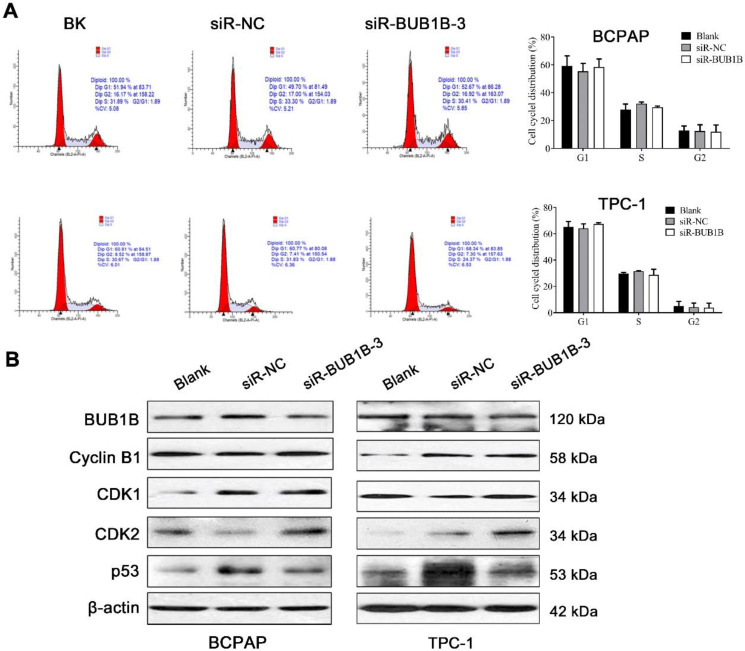
Effects of BUB1B expression on cell cycle in THCA cells. **(A)** Cell cycle in THCA cell lines with BUB1B knockdown was analyzed by flow cytometry. The two-sample *t* test was used for difference comparison between 2 groups (Blank vs siR-NC, siR-NC vs siR-BUB1B). **(B)** Western blot analysis of cell cycle-related proteins in THCA cell lines with BUB1B knockdown. Abbreviations: THCA, thyroid carcinoma; BUB1B, BUB1 mitotic checkpoint serine/threonine kinase B.

After silencing BUB1B, the THCA cell viability was significantly inhibited. From KEGG analysis, the cell cycle pathway was detected. To determine whether the above growth inhibitory phenotype was due to the disturbed cell cycle progression, we analyzed the cell cycle profile upon UBUB1B silencing by flow cytometry. However, we found no obvious influence of BUB1B knockdown on the cell cycle distribution of THCA cells (**Fig. 8A**). Cell cycle-related proteins were detected and presented as **Fig. 8B**.

**Figure 9:**
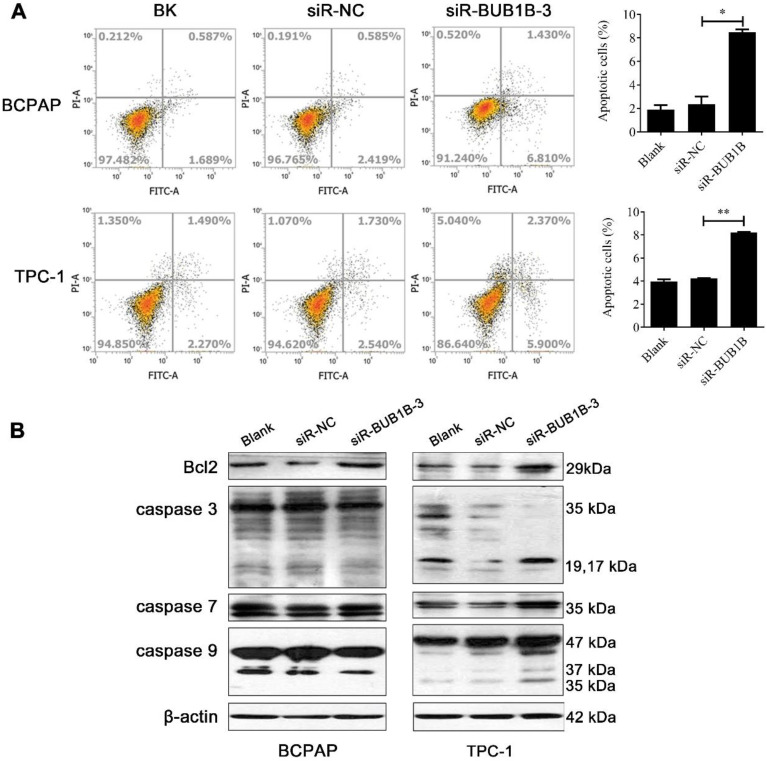
Effects of BUB1B expression on apoptosis in THCA cells. **(A)** Apoptosis of THCA cell lines with BUB1B knockdown was analyzed by flow cytometry. The two-sample *t* test was used for difference comparison between 2 groups (Blank vs siR-NC, siR-NC vs siR-BUB1B). **(B)** Western blot analysis of apoptosis-related proteins in THCA cells. Abbreviations: THCA, thyroid carcinoma; BUB1B, BUB1 mitotic checkpoint serine/threonine kinase B.

Apoptosis alteration is one of the important factors in the progression and development of cancer. We performed flow cytometry to investigate the rate of apoptosis with BUB1B knockdown. Our results displayed that the knockdown of BUB1B promoted the apoptosis of BCPAP and TPC-1 cells (**Fig. 9A**). Based on the western blotting results, the proteins related to apoptosis appeared with no obvious changes in the BCPAP cell. But apoptosis-related protein levels were elevated in TPC-1 cell such as caspase 3, caspase 7, and caspase 9. The expression of anti-apoptotic protein Bcl-2 was not influenced in 2 cells (**Fig. 9B**). It followed that BUB1B knockdown influenced the apoptosis of THCA cells.

**Figure 10:**
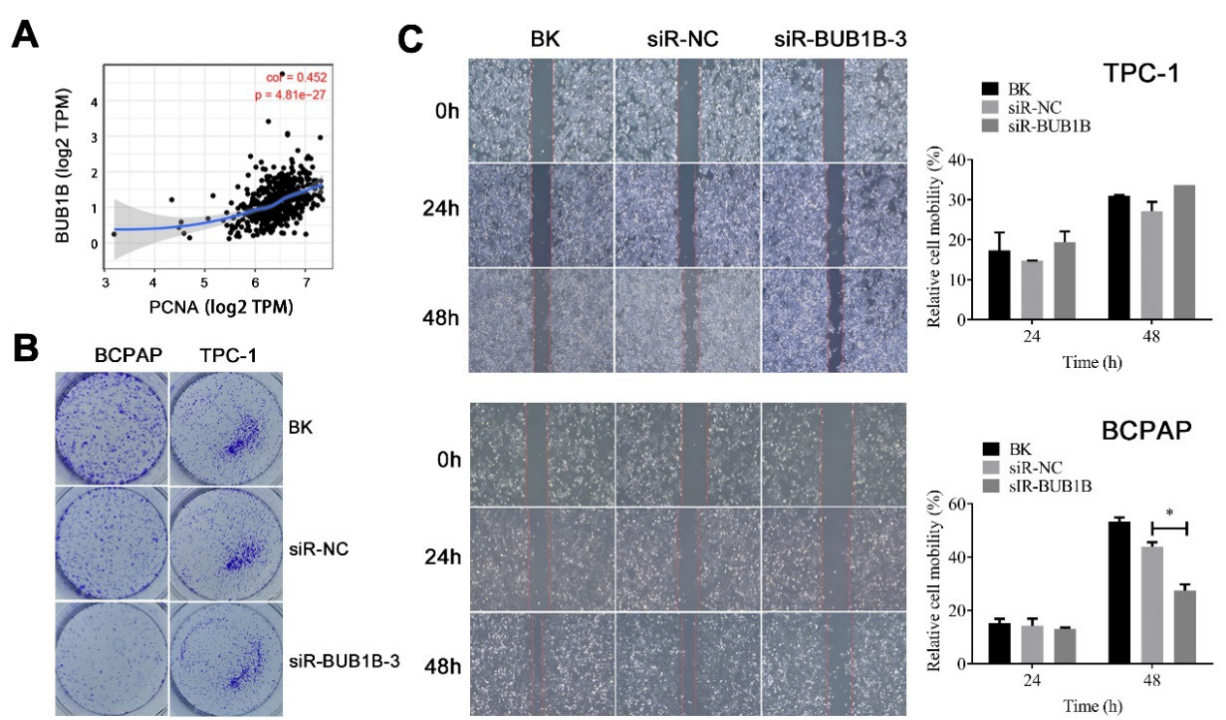
Effects of BUB1B expression on proliferation and migration in THCA cells. **(A)** Correlation between BUB1B and PCNA (TIMER). **(B)** Cell proliferation in THCA cell lines with BUB1B knockdown was assessed with colony formation. **(C)** Wound-healing assays detected cell migration in THCA cell lines with BUB1B knockdown. The two-sample *t* test was used for difference comparison between 2 groups (BK vs siR-NC, siR-NC vs siR-BUB1B). Abbreviation: PCNA, proliferating cell nuclear antigen. Abbreviations: THCA, thyroid carcinoma; BUB1B, BUB1 mitotic checkpoint serine/threonine kinase B.

We also evaluated the potential effects of BUB1B on the proliferation and migration of THCA cells. First, the relationship between BUB1B and proliferating cell nuclear antigen (PCNA) was assessed. A positive relationship between the expression of BUB1B and PCNA was noticed (**Fig. 10A**). We have observed the decreased cell viability with BUB1B knockdown. The inhibition by BUB1B knockdown was also confirmed by colony formation assay, as knockdown of BUB1B contributed to a notable decrease in the clonogenic survival of THCA cells (**Fig. 10B**). Next, we carried out a wound-healing assay for investigating the effect of BUB1B knockdown on THCA cell migration. The results suggested that the migration of BCPAP cell was significantly reduced upon BUB1B knockdown (**Fig. 10C**). No obvious inhibition was observed in TPC-1 cell. It followed that BUB1B was related to the proliferation and migration of THCA cells.

**Figure 11:**
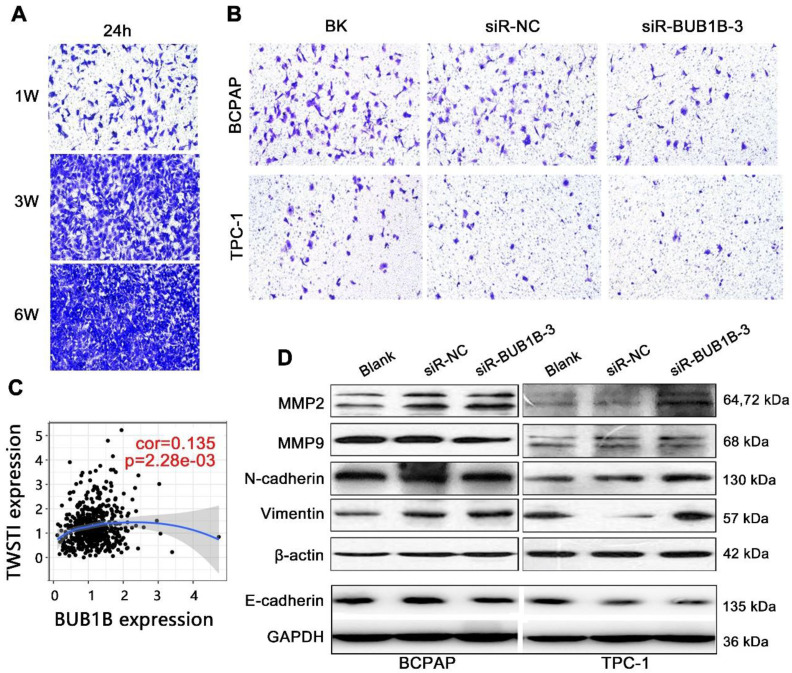
Effects of BUB1B expression on invasion and migration in THCA cells.** (A)** Cell density determination for Transwell assays. **(B)** Transwell assays detected cell migration and invasion of THCA cell lines with BUB1B knockdown.** (C)** Correlation between BUB1B and TWIST1. **(D)** Western blot analysis on migration-related proteins in THCA cell lines. Abbreviations: TWIST1, Twist Family BHLH Transcription Factor 1; THCA, thyroid carcinoma; BUB1B, BUB1 mitotic checkpoint serine/threonine kinase B.

Transwell assay was used to explore the effect of BUB1B on migration and invasion of THCA cells. The cell density of 1W was firstly identified as the proper criterion after detecting the cell migration ability (**Fig. 11A**). As determined by transwell assay, siRBUB1B treated cells have a lower invasive capacity than do control cells (**Fig. 11B**). TWIST1 can promote tumor cell invasion and metastasis, and act as a vital regulator in the EMT process. We also found a positive correlation between BUB1B and TWIST1 (**Fig. 11C**). From western blotting, MMP2, N-cadherin, and Vimentin were upregulated in the BUB1B knockdown group in 2 cells, while E-cadherin expression was down-regulated in BUB1B knockdown group (**Fig. 11D**). The change of MMP9 protein was not apparent in 2 THCA cells. These results demonstrated that BUB1B might regulate the migration and invasion of THCA cells.

### Correlation between BUB1B and cancer pathways in THCA

The above results have indicated that BUB1B might regulate the apoptosis, invasion, and migration of THCA cells. To further determine potential signaling pathways modulated by BUB1B, we performed GSEA analysis via analyzing the data from TCGA. We explored the correlation between BUB1B and migration pathways, observing that the activity of the mTOR, NF-kappa B, TGF-beta, and TNF signaling pathway was positively correlated with BUB1B expression levels in THCA tissues (**Table 7**). We have found the influence of BUB1B knockdown on cell apoptosis *in vitro*, and GSEA analysis confirmed a positive correlation between BUB1B and the apoptosis pathway. Although a significant positive regulation was observed between BUB1B and the cell cycle pathway, the disturbing effect of BUB1B knockdown on the cell cycle distribution of THCA cells was not detected in our flow cytometry assay.

## Discussion

Although THCA patients have a better clinical outcome rather than other cancer types, the patients are always subject to recurrence risk of disease. The current diagnostic biomarkers still fail to predict the progression of THCA. Therefore, it is of great interest to develop novel biomarkers for the estimation of progression and recurrence potential. Herein, we reported that BUB1B mRNA were highly expressed in THCA samples, and BUB1B expression influenced the THCA cells' growth. Overexpression of BUB1B predicted a shorter progression-free survival time of THCA. Together, these results suggested that overexpression of BUB1B presented the potential to serve as a biomarker for THCA prognosis.

The BUB1B gene is located at chromosome 15q15. The function of BUB1B in mitosis includes activation, maintenance, and silencing the SAC as well as regulating chromosome-spindle attachment, and it is also required for controlling mitotic timing. Clausen et al. found that BUB1B expression was significantly reduced in aneuploid compared to diploid cancers 22, showing that loss of spindle checkpoint function may be involved in the development of DNA aneuploidy, and thus influenced the tumor progress. Aberrant expression or mutations of BUB1B can cause aneuploidy. The abnormal high expression of BUB1B has been revealed in sarcoma 23, breast cancer 24, and hepatocellular carcinoma 25. Our study found high expression of BUB1B in THCA through bioinformatics and experimental verification *in vitro*, and BUB1B knockdown influenced the THCA cell growth. It followed that BUB1B might be involved in THCA progression. In terms of BUB1B mutation, Hanks et al. suggested that the genetic progression of BUB1B in rhabdomyosarcoma from mosaic variegated aneuploidy (MVA) and non-MVA cases may be similar, but that somatic BUB1B mutations were unlikely to be common in sporadic childhood cancers known to be associated with MVA 26. Hahn et al. indicated that germline mutations in BUB1B were at high risk for the development of early-onset colorectal cancer 27. Rio et al. reported that a germline homozygous intrinsic mutation of BUB1B increased the susceptibility to gastrointestinal oncogenesis 28. It followed that BUB1B mutation was significantly related to tumor development. In this study, we observed the genetic alteration of BUB1B in THCA, indicating the potential influence on THCA development. Several mutations of BUB1B were associated with cancers such as M40T in colorectal cancer and Q363R in breast cancer 29. However, we did not find the mutation site of BUB1B in THCA samples. BUB1B mutation not only correlated with tumor progression but also was detrimental to woman's health. Chen et al. found that heterozygous deleterious variants of BUB1B can be able to induce premature ovarian insufficiency and early menopause 30. Apart from abnormal expression and mutation, polymorphisms in BUB1B may contribute to tumorigenesis and the risk of tumor development 31.

To further assess the importance of BUB1B in clinical, we explored the effect of BUB1B expression level on the prognosis of THCA patients. We found that BUB1B high expression shortened the progression-free survival time of THCA patients, but did not influence the patient's overall survival. High expression of BUB1B increased the recurrence risk of THCA patients. BUB1B has been identified as an immune-related gene in prostatic adenocarcinoma, acting as a component of risk signature, and exhibiting a good prognostic value and predictive accuracy 32. BUB1B was also involved in the molecular regulation of EMT and associated with poor early survival of ovarian serous carcinoma at stages I+II 33, indicating BUB1B as a key biomarker for early clinical diagnosis and prognosis evaluation of patients. Jiang et al. constructed a model using 10 cell cycle-related genes including BUB1B, finding that signature could precisely predict the prognosis of lung adenocarcinoma patients 34. These researches have presented the vital prognostic value of BUB1B in human cancers and revealed its potential as a useful therapeutic target. In this study, BUB1B was also proved to be an independent prognostic factor for PFS of THCA patients. The result further demonstrated the importance of BUB1B in THCA. It was worth mentioning that, we did not find a favorable prognostic impact of BUB1B high expression among 33 cancer types by pan-cancer analysis. Worse was that BUB1B was highly expressed in almost all cancers. It suggested that BUB1B might act as an oncogene and significantly affect the clinical outcome of patients.

From KEGG analysis for revealing the potential mechanism of BUB1B, we found that BUB1B was significantly related to the cell cycle pathway. However, we did not find an obvious disturbing effect of BUB1B on cell cycle distribution by flow cytometry in this study, and cell cycle-related proteins showed no apparent changes. Fu et al. found that BUB1B overexpression enhanced the proliferation, migration, and invasion ability of prostate cancer cells, whereas depletion of BUB1B did not affect the cell functions 35. But our study conducted BUB1 knockdown and found that BUB1B knockdown inhibited the proliferation, migration, and invasion of THCA cells. GSEA analysis indicated that BUB1B was positively correlated with metastasis-related pathways such as mTOR and NF-kappa B pathways. Qiu et al. found that BUB1B promoted hepatocellular carcinoma progression via activating the mTORC1 signaling pathway, and the oncogenic effect of BUB1B can be impaired when the mTORC1 signaling pathway was inhibited 25. Harima et al. pointed that BUB1B was associated with tumor metastasis via chromosomal instability 36. Several studies have suggested some useful biomarkers targeting BUB1B. By targeting BUB1B via shRNAs, BUB1B-dependent radioresistance can be decreased in glioblastoma 37. Wan et al. has indicated that BUB1B was a direct transcriptional target of Forkhead Box M1 (FOXM1), and suppression of FoxM1 could reduce BUB1B expression and inhibit cell growth and survival 38. Mechanistically, FOXM1 transcriptionally regulated BUB1B expression by binding to and then activating the BUB1B promoter 37. He et al. found that miR-210 could suppress the expression of BUB1B by directly targeting its 3'-UTRs, and over-expression of exogenous miR-210 disturbed mitotic progression and caused aberrant mitosis 39. It was highly necessary to detect more promising biomarkers targeting BUB1B, which was conducive to inhibit disease progression and improve patient's clinical outcomes.

This study revealed the function of BUB1B in THCA, revealing that BUB1B overexpression was unfavorable to the patient's progression-free survival, and BUB1B knockdown affected the proliferation, invasion, and migration of THCA cells. But we failed to further confirm its function through other methods such as overexpression analysis. We did not find the disturbing effect of BUB1B on the cell cycle distribution of THCA cells, which was unexpected for us as BUB1B is a known cell cycle-related gene. Therefore, more verification methods were needed to detailly reveal the function of BUB1B. This is one of the limitations of this study. In addition, we predicted several metastasis-related pathways, and it will be better if the experimental verifications are further conducted.

## Conclusions

In this study, the high expression of BUB1B in THCA tissues was confirmed by bioinformatic analysis and experimental verification *in vitro*. We found that BUB1B knockdown influenced the THCA cell growth and BUB1B high expression predicted a shorter progression-free survival, which suggested that BUB1B might be involved in THCA progression. KEGG analysis initially predicted that BUB1B was related to the cell cycle pathway, but a disturbing effect on THCA cells caused by BUB1B knockdown was not detected in our study. However, BUB1B knockdown influenced the proliferation, invasion, and migration of THCA cells. We further predicted a positive correlation between BUB1B and metastasis-associated pathways. It should be noted that BUB1B might be also involved in THCA development by combining other important prognostic biomarkers. Our results revealed the oncogenic role of BUB1B in THCA through series of bioinformatics and functional assays.

## Figures and Tables

**Table 1:** Three differential small interfering RNA sequences.

siRNA	sense	antisense
siR-NC	UUCUCCGAACGAGUCACGUTT	ACGUGACUCGUUCGGAGAATT
siR-BUB1B-1	CUGUAUUGUUUGGCACCAAUATT	UAUUGGUGCCAAACAAUACAGTT
siR-BUB1B-2	GAGACAACUAAACUGCAAAUUTT	AAUUUGCAGUUUAGUUGUCUCTT
siR-BUB1B-3	CCAGUUCUGUUUGUCAAGUAATT	UUACUUGACAAACAGAACUGGTT
siR-TOP2A-1	ACUGUGGGCGCAUUGUAAGGGTT	UACAUAAUCAACAUGUCUGCCTT
siR-TOP2A-2	AGCUCUUUGGCUCGAUUGUUATT	UAACAAUCGAGCCAAAGAGCUTT
siR-TOP2A-3	AGGCGUUUGAUGGAUGGAGAATT	UUCUCCAUCCAUCAAACGCCUTT

**Table 2:** List of antibodies used

protein name	Company	Art. No.	Protein name	Company	Art. No.
BUbR1(BUB1B)	Abcam	ab183496	CDK2	Abcam	ab32147
TOP2A	Abcam	ab52934	P53	CST	2525
CDK1	Abcam	ab133327	CyclinB1	Abcam	ab32053
Bcl-2	CST	15071	Caspase-3	Abcam	ab13847
Caspase-9	CST	9502	Caspase7	Proteintech	271555-1-AP
MMP2	CST	87809S	MMP9	Abcam	ab76003
Vimentin	CST	5741	N-cadherin	Proteintech	22018-1-AP
Goat anti-Mouse IgG Antibody	CST	7076	Goat anti-Rabbit IgG Antibody	CST	7074
β-actin	CST	4970	E-cadherin	CST	3195
GAPDH	CST	5174			

**Table 3:** The clinical characteristics of THCA patients based on TCGA data

Parameters	Number	Subtypes	Low	High	χ^2^	P
person neoplasm cancer status	n=435	tumor free	201	199	2.212	0.160
		with tumor	13	22		
age	n=504	≤55	176	178	0.038	0.922
		>55	76	74		
gender	n=504	male	67	71	0.160	0.764
		female	185	181		
T stage	n=502	T1	74	67	3.747	0.290
		T2	90	75		
		T3	78	96		
		T4	10	12		
N stage	n=455	N0	124	103	6.172	0.015
		N1	98	130		
M stage		M0	140	146	0.003	0.953
n=294		M1	4	4		
clinical stage	n=502	Stage I	132	152	11.172	0.011
		Stage II	37	15		
		Stage III	56	55		
		Stage IV	25	30		
radiation therapy	n=448	no	85	84	0.039	0.846
		yes	143	136		
neoplasm depth	n=403	≤1	46	57	0.684	0.710
		1-2	74	77		
		≥2	74	75		
neoplasm length	n=475	≤3	154	145	1.564	0.457
		3-6	68	74		
		≥6	14	20		
neoplasm width	n=425	≤2	105	119	4.586	0.101
		2-4	83	70		
		≥4	18	30		
progression-free survival status	n=504	without recurrence	237	214	11.154	0.001
		recurrence	15	38		
overall survival status	n=504	alive	243	245	0.258	0.800
		dead	9	7		

**Table 4:** Kaplan-Meier analysis in terms of PFS on BUB1B in THCA

		Low(N=192)	High (N=192)	HR (95%CI)	Log-rank P
clinical stage	I	142	142	3.09 (1.12-8.50)	0.02
	II	26	26	0.79 (0.13-4.75)	0.80
	III	55	56	1.83 (0.66-5.05)	0.23
	IV	27	28	4.65 (1.19-18.13)	0.02
gender	male	69	69	2.08 (0.83-5.22)	0.11
	female	183	183	3.88 (1.68-8.94)	0.0061
age	≤55	177	177	2.64 (1.16-5.99)	0.02
	>55	75	75	2.94 (1.23-7.06)	0.01

Abbreviation: THCA, thyroid carcinoma; BUB1B, BUB1 mitotic checkpoint serine/threonine kinase B; PFS, progression-free survival; HR, hazard ratio; CI, confidence interval; N, sample number.

**Table 5:** Cox regression analysis on BUB1B in THCA patients based on TCGA data

Variables	PFS		OS	
	HR (95% CI)	P	HR (95% CI)	P
Univariate				
BUB1B	2.55 (1.52-4.28)	<0.001	0.77 (0.16-3.82)	0.752
age	1.02 (1.00-1.04)	0.028	1.16 (1.10-1.22)	<0.001
gender	0.58 (0.33-1.01)	0.053	0.53 (0.19-1.46)	0.220
T stage	1.85 (1.35-2.53)	<0.001	2.63 (1.42-4.88)	0.002
N stage	1.59 (0.90-2.83)	0.113	1.45 (0.47-4.44)	0.516
M stage	5.88 (2.04-17.01)	0.001	5.45 (1.16-25.57)	0.031
Clinical stage	1.53 (1.22-1.92)	<0.001	2.42 (1.54-3.82)	<0.001
Multivariate				
BUB1B	2.93 (1.10-7.81)	0.032	1.324 (0.11-15.79)	0.825
age	1.01 (0.98-1.05)	0.220	1.18 (1.07-1.30)	0.001
gender	1.12 (0.50-2.47)	0.774	0.85 (0.14-4.93)	0.856
T stage	1.26 (0.73-2.19)	0.399	2.41 (0.38-15.20)	0.349
N stage	0.92 (0.41-2.08)	0.855	0.47 (0.05-3.98)	0.492
M stage	2.51 (0.63-10.03)	0.191	4.54 (0.33-62.14)	0.257
Clinical stage	1.29 (0.74-2.23)	0.358	0.84 (0.11-6.08)	0.864

Abbreviations: THCA, thyroid carcinoma; BUB1B, BUB1 mitotic checkpoint serine/threonine kinase B; HR, hazard ratio; CI, confidence interval; PFS, progression-free survival; OS, overall survival.

**Table 6:** Cox regression analysis on TOP2A in terms of PFS in THCA patients

Variables	Univariate analysis		Multivariate analysis	
	HR (95% CI)	P	HR (95% CI)	P
TOP2A	1.506 (1.194-1.901)	0.001	1.512 (1.195-1.914)	0.001
age	0.993 (0.971-1.016)	0.545	0.999 (0.972-1.027)	0.942
stage	0.901 (0.647-1.254)	0.536	0.896 (0.598-1.341)	0.896
gender	1.017 (0.477-2.171)	0.965	0.990 (0.462-2.119)	0.979

Abbreviation: THCA, thyroid carcinoma; BUB1B, BUB1 mitotic checkpoint serine/threonine kinase B; TOP2A, Topoisomerase (DNA) II Alpha; PFS, progression-free survival; HR, hazard ratio; CI, confidence interval;

**Table 7:** Correlation of BUB1B with important cancer-related pathways involved in THCA by GSEA analysis

Pathway name	NES	P
NF-kappa B signaling	1.9566	0.0019
TGF-beta signaling	1.7434	0.0195
Apoptosis	2.1453	<0.0001
mTOR signaling	1.8421	0.0035
Cell cycle	2.4330	<0.0001
TNF signaling	1.9001	0.0034

Abbreviation: THCA, thyroid carcinoma; BUB1B, BUB1 mitotic checkpoint serine/threonine kinase B; GSEA, gene set enrichment analysis; NES, normalized enrichment score; NF, nuclear factor; TGF, transforming growth factor; mTOR, mammalian target of rapamycin; TNF, tumor necrosis factor.

## Abbreviations

THCA: thyroid carcinoma
BUB1B: BUB1 mitotic checkpoint serine/threonine kinase B
KEGG: Kyoto Encyclopedia of Genes and Genomes
GSEA: gene set enrichment analysis
PFS: progression-free survival
OS: overall survival
mTOR: mammalian target of rapamycin
NF-kappa B: nuclear factor kappa-B
GSCA: Gene Set Cancer Analysis
TOP2A: Topoisomerase (DNA) II Alpha
BP: biological process
CC: cellular component
MF: molecular function
OD: optical density
